# Quail egg homogenate alleviates food allergy induced eosinophilic esophagitis like disease through modulating PAR-2 transduction pathway in peanut sensitized mice

**DOI:** 10.1038/s41598-018-19309-x

**Published:** 2018-01-18

**Authors:** Priscilia Lianto, Shiwen Han, Xinrui Li, Fredrick Onyango Ogutu, Yani Zhang, Zhuoyan Fan, Huilian Che

**Affiliations:** 1 Beijing Advanced Innovation Center for Food Nutrition and Human Health, College of Food Science and Nutritional Engineering, China Agricultural University, Beijing, 100083 P.R. China; 2 College of Food Science and Nutritional Engineering, China Agricultural University, Beijing, 100083 P.R. China; 3 Food Technology Division of Kenya Industrial Research and Development Institute, South C – Popo Rd., Off Mombasa Rd., 30650–00100 Nairobi, Kenya

## Abstract

The present pharmacotherapy for eosinophilic esophagitis (EoE) fundamentally depend on inhaled corticosteroids. Despite the fact that oral intake of topical steroids can be successful in restricting EoE-related inflammation, there are concerns with respect to the long term utilization of steroids, especially in kids. In the current research, we assess the effect of quail egg, which is reportedly a known serine protease inhibitor, on symptomatology and immune responses in a peanut-sensitized mouse model of food allergy induced EoE. Daily oral treatment with quail egg attenuated mice symptomatology and immune response. Treatment with quail egg inhibited antigen-prompted increments in mouse tryptase and eosinophil cationic protein (ECP) in serum and eosinophil in inflamed tissues like oesophagus, lung, and digestive system. Quail egg treatment resulted in decreased antibody specific IgE and IgG1 and a variety of inflammatory genes that were abnormally expressed in EoE. Other effects included increased IL-10, decreased PAR-2 activation and NF-kB p65 in inflamed tissues. Our results suggest that quail egg treatment may have therapeutic potential in attenuating the symptoms of food allergy induced EoE like disease through regulating PAR-2 downstream pathway by blocking the activation of the transcription factor NF-kB p65 activity.

## Introduction

Eosinophilic esophagitis (EoE) is a chronic inflammatory disease associated with food allergy^1–3^ which seriously affects the patient’s normal life. This disease manifests in the patient’s oesophagus which characterized by an endless inflammatory state. In Western countries, the prevalence of EoE has increased dramatically from 10 to 50 for every 100,000 general population^4^. Meanwhile, albeit published reports of EoE from Asian countries are restricted. Kinoshita *et al*. conducted a recent systematic literature review of EoE in Asian countries which revealed comparative EoE illness pathogenesis amongst Western and Asian patients^5^. The prevalence of EoE in Asian countries is nearly 17–6,557 per 100,000 endoscopy investigated cases, presenting a wide range potentially attributable to an implicit study bias as the effect of using a small sample size and or different forms of endoscopy indication. Even though reported prevalence rates of EoE in Asian population have been greatly variable, the characterisrics of EoE disease pathogenesis were similar to those found in Western population. As a matter of fact, EoE has now turned out to be serious diseases not only in Western countries but also in Asia^5^.

As of now, the essential pharmacotherapy for EoE fundamentally depend on inhaled corticosteroids. In spite of the fact that oral intake topical steroids can be successful in constraining EoE-related inflammation, there are concerns with respect to the long term utilization of steroids, for instance, oropharyngeal, oesophageal candidiasis, bone mineral density abnormalities, glaucoma, hyperglycaemia, and other antagonistic impacts^2,6–8^. There is a pressing need to find and create novel and powerful anti-inflammatory drugs for EoE.

It was revealed in a previous study that quail egg is different from other bird eggs. The difference is particularly in its egg white, quail egg is richer in proteins that have anti-allergic and anti-inflammatory effects^9^. *In vitro* study found that quail egg contain ovomucoid and ovoinhibitor that can alleviate allergic reactions by blocking the binding of tryptase or any other trypsin homolog and protease-activated receptor 2 (PAR-2)^10^. Moreover, a patent on the role of quail egg in modulating the immune cell function, particularly eosinophils and neutrophils, in the treatment of allergy was issued in 2015 by the United States (patent number: US2015/0057232A1)^11^, giving solid foundations of the special role of quail eggs.

The role of quail egg has been determined in the context of allergic asthmatic^12,13^ and rhinitis^14^ treatments, but not in food allergy, particularly its role in the treatment of food allergy induced EoE like disease has not been reported previously. Therefore, this study used mouse model of peanut allergen induced EoE like disease to study the anti-inflammatory and anti-allergic effects of quail egg and its mechanism in treating food allergy.

## Results

### Oral quail egg treatment relieved the symptoms of EoE-like food allergy disease in Balb/c mice

**Figure 1:**
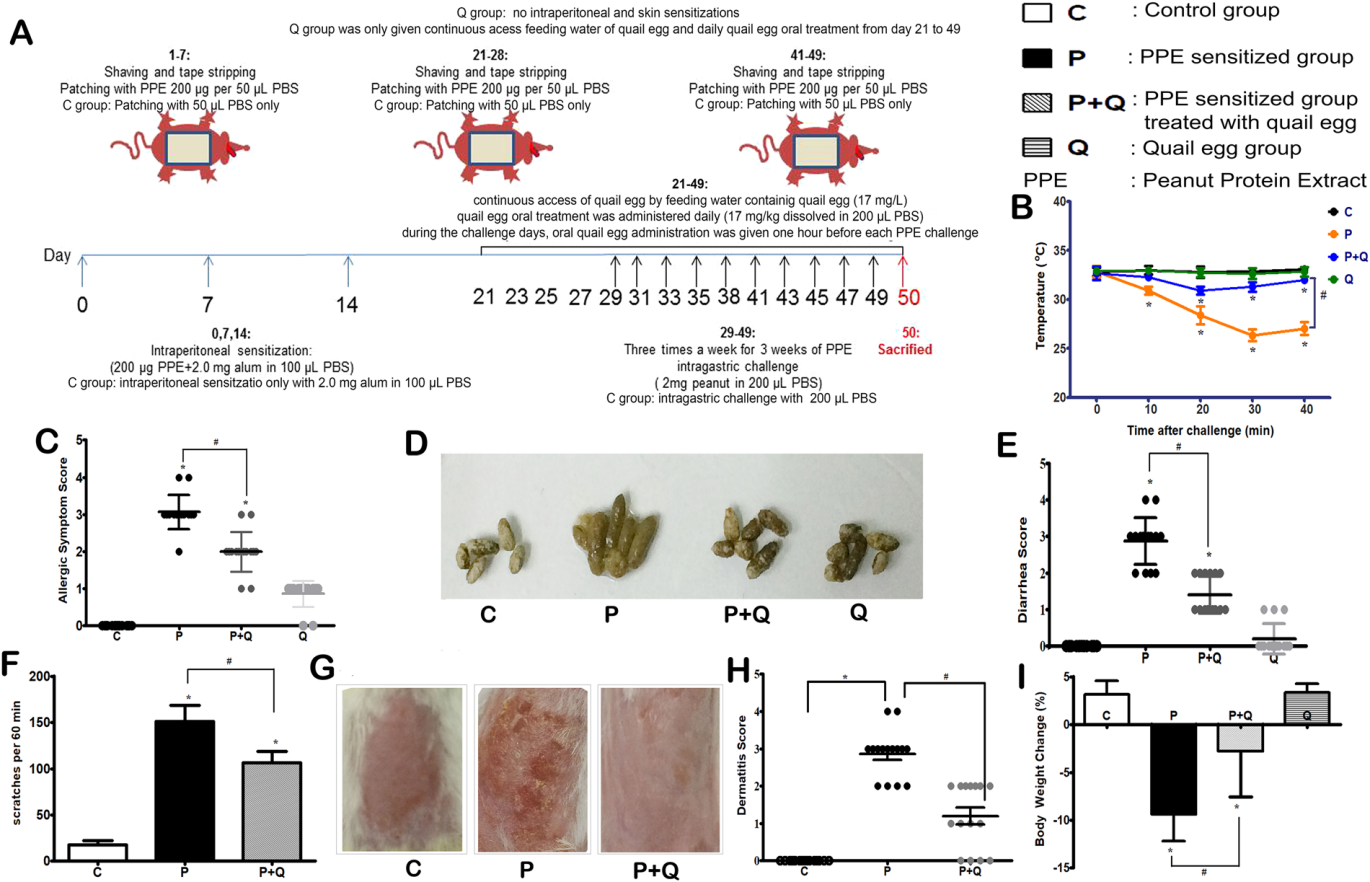
Oral quail egg treatment alleviated Balb/c mice food allergy induced EoE like disease symptoms (n = 15). (**A**) Protocol for the peanut protein extract (PPE) induced EoE like food allergy model; (**B**) body temperature change; (**C**) hypersensitivity response; (**D**) Mice stools; (**E**) Diarrhoea scores; (**F**) numbers of scratches per 60 min; (**G**) Mice rostral part of the back; (**H**) Dermatitis scores; (**I**) Body weight change. Results are expressed as mean ± SEM. *P < 0.05 as compared to C group, ^#^*P* < 0.05 as compared to P group.

The occurrence of food allergy is usually indicated by systemic and local allergic symptoms and/or signs^15^. In order to determine whether the quail egg could alleviate the food allergy induced EoE like disease symptoms, we conducted observations on the systemic (body temperature and hypersensitivity response) and local allergic reactions (diarrhoea, scratching behaviour, dermatitis, and body weight loss) of mice within 40 min after skin or intragastric challenge. Protocol of peanut allergens induced EoE like disease in murine model is described in Fig. 1A.

The protein peanut extract (PPE) sensitized group (P group) body temperature dropped to the lowest point within 40 min after challenge. Compared to control group (C group), the body temperature of P mice group dropped by 6.50 ± 0.76 °C (*P* < 0.05) while after giving oral quail egg treatment, the drop observed in PPE sensitized group treated with quail egg (P + Q group) was lower, with a drop of 2.00 ± 0.56 °C (*P* < 0.05). Independent treatment with quail egg (Q group) did not show the change in body temperature (Fig. 1B). The scores of clinical allergy symptoms observed in mice were consistent with the decrease of body temperature. The score of allergic symptoms in P group was 3.07 ± 0.46, while after oral quail egg treatment, the allergic symptoms as observed in P + Q group were decreased to 2.00 ± 0.53 *(P* < 0.05, Fig. 1C) There was no significant difference between mice treated with quail egg alone and control group (C: 0.26 ± 0.34 vs Q: 0.87 ± 0.35).

Repeated oral allergen challenges triggered the occurrence of diarrhoea symptom in P group mice (2.87 ± 0.64), while oral treatment with quail egg significantly inhibited the occurrence of diarrhoea in P + Q group mice (1.40 ± 0.51) indicated by amelioration change of watery stool. Meanwhile, mice treated with quail egg alone (Q group) did not show diarrhoea response (1.00 ± 0.85, Fig. 1D–E). In addition to skin response, we recorded mice scratching behaviour within 60 min after antigen skin stimulation and also the severity degree of dermatitis in back skins of mice were also evaluated (Fig. 1F–H). Q group was developed in order to establish whether giving oral quail egg treatment in the healthy mice could elicit allergic responses, therefore, in the current study we did not conduct either intraperitoneal or skin sensitizations, but conducted oral quail egg administration to mice in Q group. Hence, we did not conduct the analysis on skin responses in the Q group. Mice in P group showed 192.53 + 40.01 per 60 min scratches which developed to the skin dermatitis score level of 3.13 ± 0.74. Oral treatment with quail egg was significantly able to reduce the scratching symptom 103.27 ± 25.24 scratches per 60 min) and dermatitis score (1.20 ± 0.86) of P + Q group (*P* < 0.05). Moreover, we recorded the weight loss of mice between one day before the first challenge (day 28) and the last challenge (day 49) as shown in Fig. 1I. As compared to control group, repeated PPE stimulation significantly decreased P group body weight (−9.40 ± 2.75%, *P* < 0.05) while oral quail egg treatment was able to relieve mice (P + Q group) weight loss (−2.81 ± 4.76%, *P* < 0.05). Mice treated with quail egg alone (Q group) showed no significant weight loss (3.37 ± 0.87%).

From the body weights, systemic and local allergic reaction results, as shown in Fig. 1, we might identify the effect of oral quail egg treatment in alleviating PPE induced EoE like food allergy disease symptoms.

### Oral quail egg treatment reduced the levels of PPE specific IgE, IgG1 and allergic mediators

**Figure 2:**
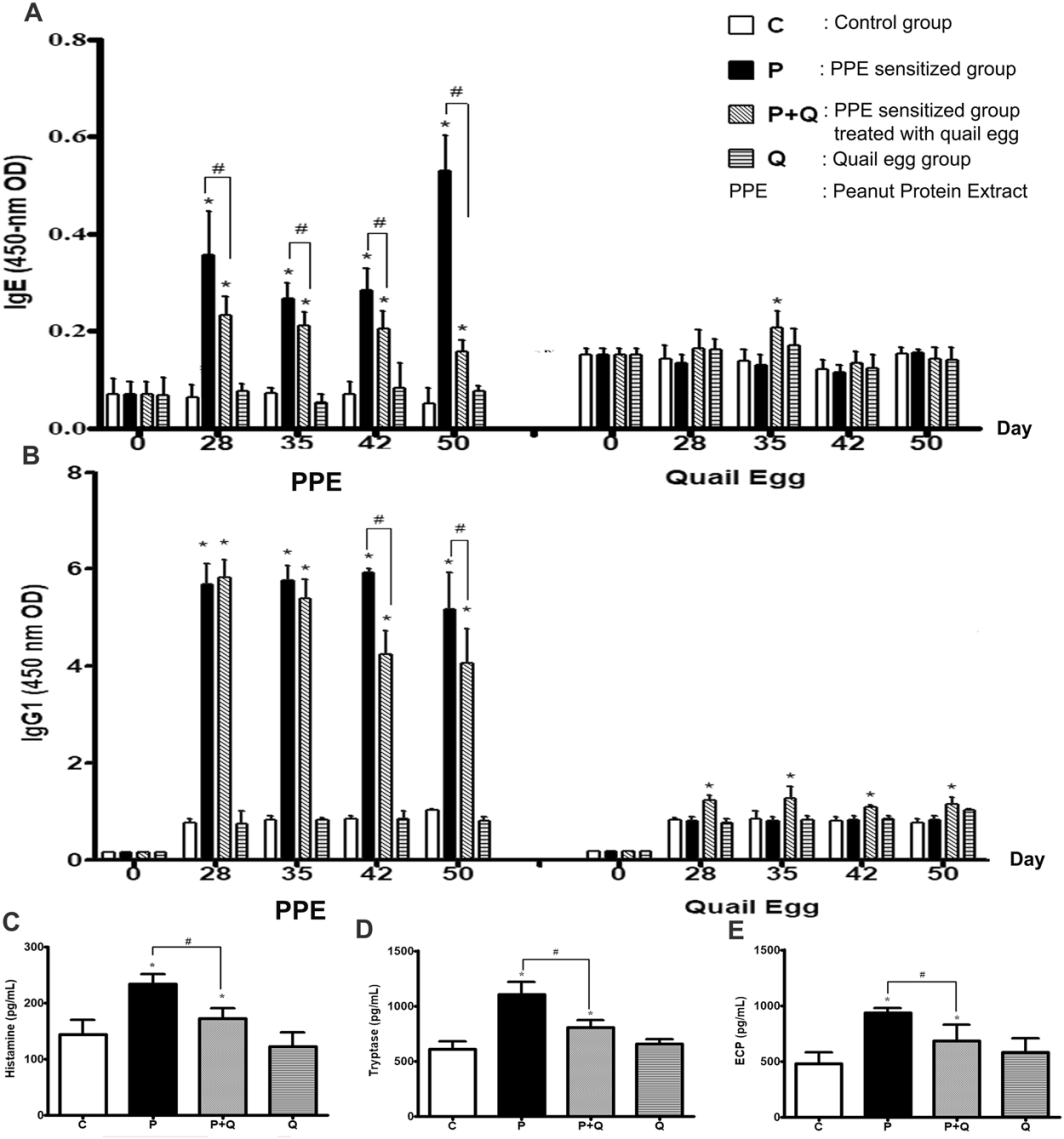
Oral quail egg treatment reduced PPE specific IgE, IgG1, and the level release of allergic mediators (n = 10). (**A**) PPE and quail egg specific IgE levels (**B**) PPE and quail egg specific IgG1 levels; Allergic mediators: (**C**) histamine, (**D**) tryptase; (**E**) ECP. Results are expressed as mean ± SEM. *P < 0.05 as compared to C group, ^#^*P* < 0.05 as compared to P group.

Allergen specific IgE and IgG1 belong to type Th2 type antibodies^16^, it can reflect the immune condition of the body. Around 80% of the considerable number of antibodies in the body is IgG and a trace amount is IgE^17^. Due to the fact that quail egg can also allow the body to generate specific antibodies, we conducted analysis on peanut and quail egg specific IgE and IgG1 antibody levels. As shown in Fig. 2A, in the beginning of PPE challenge (day 28), the level of PPE specific IgE in P group was significantly increased as compared to control group (*P* < 0.05). Following PPE repeated challenge within day 29 to 49, the level of PPE specific IgE decreased, and reached the highest level on the last day of experiment (day 50). Daily administration of quail egg was able to significantly reduce the level of PPE specific IgE as observed in P + Q mice group (*P* < 0.05) whereas mice treated with quail egg alone did not generate PPE specific IgE. Meanwhile, during the experimental period, there was no significant quail egg specific IgE level changes found in each group. However, on the day 35 of experiment, the significant increase of quail egg specific IgE was observed in P + Q group and Q group (*P* < 0.05). The absorbance of PPE specific IgE and quail egg specific IgE in P + Q group shown similar level which were 0.27 ± 0.03 and 0.21 ± 0.02, respectively.

Moreover, the change of IgG1 level is normally consistent with the change of IgE level. As shown in Fig. 2B, during the sensitization phase (day 28), P group showed high levels of PPE specific IgG1, the high IgG1 level continued until the end of the experiment (*P* < 0.05) which was consistent with allergic response or activation of mast cells. As compared to day 0, each quail egg specific IgG1 was increased in all P + Q group during experiment period, the increase of quail egg specific IgG1 was statistically significant (*P* < 0.05). Meanwhile, following oral quail egg treatment, P + Q group showed a significant decreased of PPE specific IgG1 levels in the last two weeks of challenge. The results shown in Fig. 2B indicated that although daily oral quail egg treatment was able to inhibit the production of PPE specific IgG1, oral quail treatment alone was on the other hand able to stimulate production of quail egg specific IgG1 in P + Q group, which was consistent with the augment quail egg specific IgE detected on day 35. These findings indicated that peanut protein extract and quail egg together were able to mount a response against the two antigenic sources in P + Q mice group.

Mast cells and eosinophils activation play an important role in allergic reactions^18,19^. Therefore, in order to determine the degree of mast cell and eosinophil activation in experimental mice, we conducted analysis on allergic mediators released in serum as well as histamine, tryptase, and eosinophil cationic protein (ECP) levels (Fig. 2C-E). As compared to control (C group), these three allergic mediators were significantly increased in P group whereas oral quail egg treatment (P + Q group) was able to significantly inhibit the release of these three mediators (*P* < 0.05), whereas we found no significant difference between C and Q group. It is known that reduction of mast cells and eosinophils is consistent with decreased allergy^20^. Eosinophils are central effector cells in allergy which increases allergic inflation^20^, histamine are effector molecules released on during allergenic response, the two are key components of allergic response, where their quantities are increased but when allergy is modulated, their quantity is reduced^20^.

### Oral quail egg treatment reduced spleen cells released EoE related cytokines

**Figure 3:**
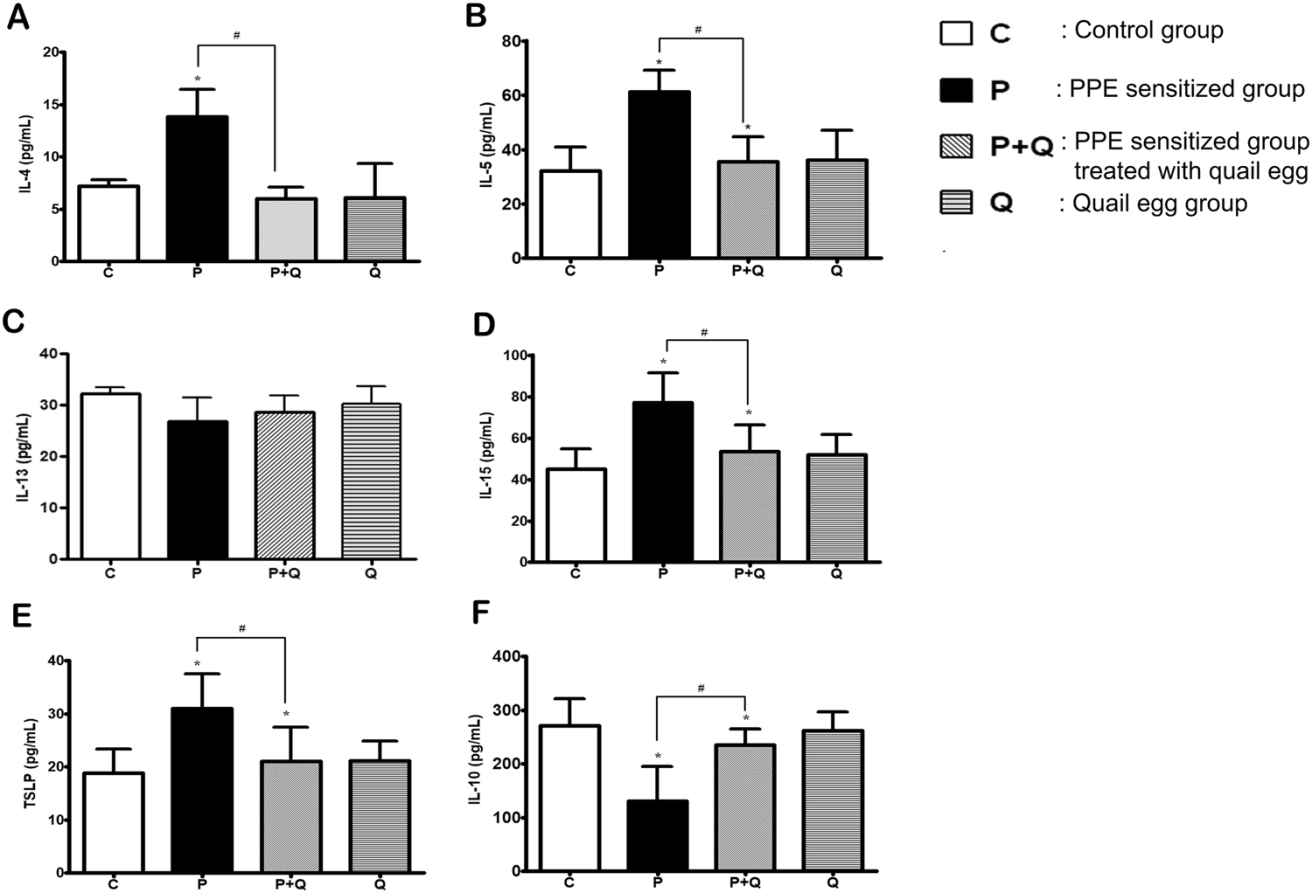
Oral quail egg treatment reduced spleen cells released EoE related cytokines (n = 5). Th2 cytokines (**A**) IL-4, (B) IL5, (**C**) IL-13; iNKT cytokines (**D**) IL-15; Atopic dermatitis related cytokine (E) TSLP; Allergic negative regulators (**F**) IL-10. Results are expressed as mean ± SEM. *P < 0.05 as compared to C group, ^#^*P* < 0.05 as compared to P group.

Clinical studies in mice and humans revealed the role of Th2 cells and iNKT-cells in the disease pathogenesis^21^. We, therefore, investigated the effect of quail egg treatment on Th2-cytokines (IL-4, IL-5, and IL-13), negative regulator (IL-10), iNKT cell cytokine (IL-15), and atopic dermatitis related cytokine (TSLP). Compared to control group, PPE sensitized and challenged mice (P group) showed a significant increase in Th2 IL-4 and IL-5 cytokines (*P* < 0.05). Following oral quail treatment, the levels of IL-4 and IL-5 of P + Q group was restored to the normal level whereas mice treated with quail egg alone did not affect the production of IL-4 and IL-5 cytokines (Fig. 3A–B). We observed that IL-13 was not affected by either PPE sensitization or quail egg treatment (Fig. 3C). In addition to IL-15 and TSLP (Fig. 3D–E), we found the same line result with the changes of Th2 (IL-4 and IL-5) cytokines, PPE challenge also induced the reduction of IL-10 production (*P* < 0.05) while quail egg treatment was able to up-regulate the production of IL-10 (Fig. 3F).

### Oral quail egg treatment reduced eosinophil numbers and its related mediators

Studies have reported that food allergens could infiltrate the tissues within organs, stimulate epithelial cells to release pro-inflammatory cytokines, and thus facilitate the subsequent eosinophil activation as well as supporting the development of EoE like disease in allergic patients^2,21,22^. A recent animal model food allergy induced EoE disease study revealed that oral peanut allergen challenge might promote the accumulation of eosinophil infiltration in oesophagus, lung and small intestine^21^. Therefore, we conducted further analysis to determine the effect of oral quail treatment on modulating eosinophil infiltration during EoE disease progression.

**Figure 4:**
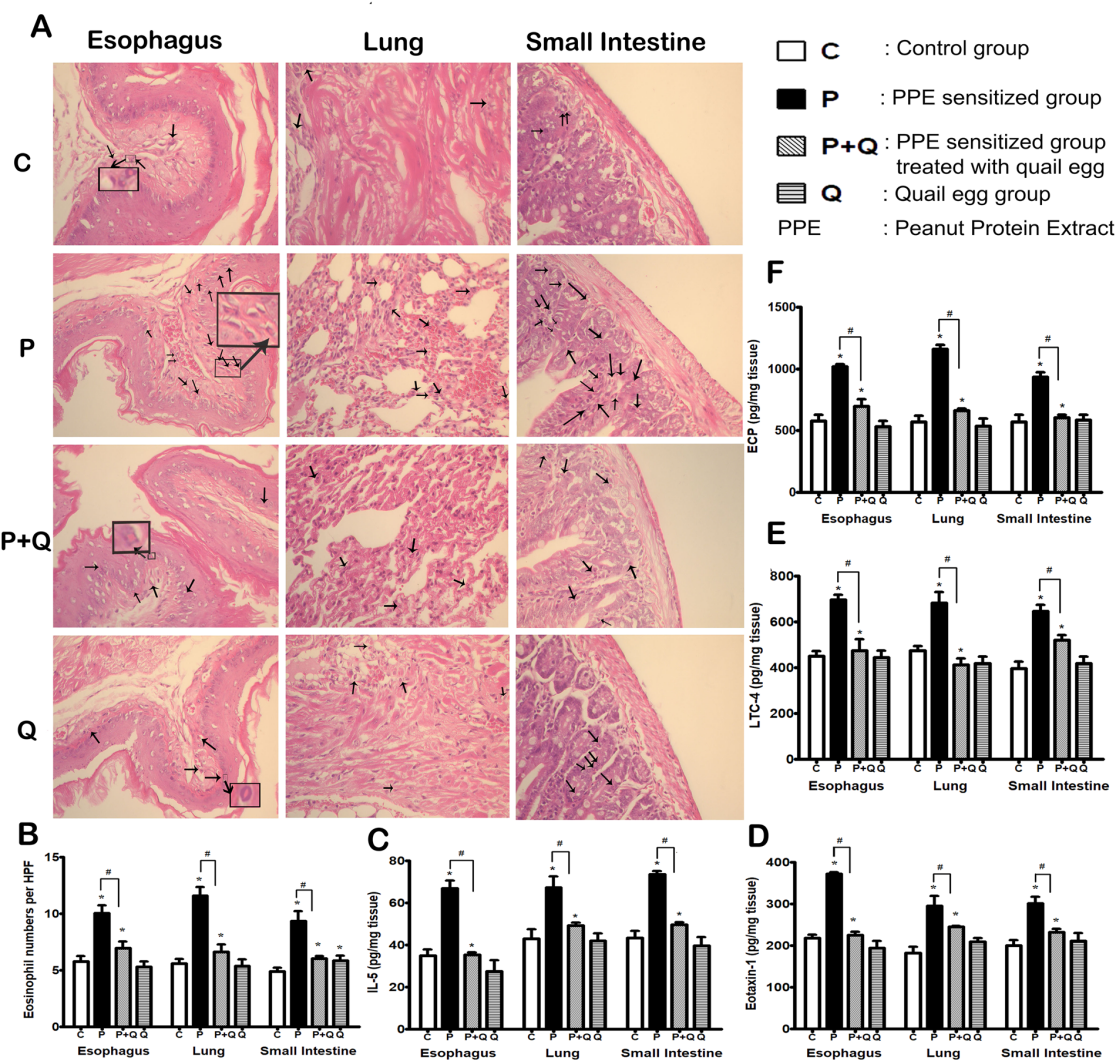
Oral quail egg treatment reduced eosinophil numbers and its related mediators (n = 5). (**A**) H&E Staining; (**B**) The numbers of Eosinophils, Scale bar 100 μm at 400×; mediators released in organ tissues: (**C**) IL-5; (D) eotaxin-1; (**E**) LTC_4_; (**F**) ECP levels. Results are expressed as mean ± SEM. *P < 0.05 as compared to C group, ^#^*P* < 0.05 as compared to P group.

First, the numbers of eosinophil infiltration in the oesophagus, lung and small intestine were counted at high magnification. Results showed a significant increase in the number of eosinophils in the three inflamed tissues of P group (*P* < 0.05, oesophagus: (10.04 ± 1.57) × 10^2^, lung: (11.60 ± 1.70) × 10^2^, small intestine: (9.34 ± 1.96) × 10^2^ per mm^2^ tissue layers). Oral quail egg treatment significantly reduced the numbers of eosinophil infiltration as observed in P + Q group (*P* < 0.05, oesophagus: (6.97 ± 1.26) × 10^2^, lung: (6.62 ± 1.46) × 10^2^, small intestine: (6.05 ± 0.48) × 10^2^ per mm^2^ tissue layers) whereas quail egg treatment of no PPE sensitized mice (Q group), except in small intestine ((5.87 ± 0.95) × 10^2^ per mm^2^ tissue layers), did not show an increase of eosinophil numbers as compared to the numbers of eosinophil infiltration in small intestine of control group ((5.688 ± 1.34) × 10^2^ per mm^2^ tissue layers) (Fig. 4A–B).

At inflammatory foci, allergen-driven recruitment of eosinophils occurs through critical mediators like IL-5 and eotaxin-1 family of chemokines^23^ which induce the activation of eosinophils. In this site of inflammation, eosinophils get activated; induce synthesis of lipid mediators like leukotriene (LTC4) and toxic granule proteins such as eosinophil cationic protein (ECP), leading to tissue damage, and triggering allergic inflammation symptoms^24^. Therefore, we assessed the expression of these eosinophil related mediators released in tissues. PPE allergen challenge induced the elevation of IL-5 (Fig. 4C), eotaxin-1 (Fig. 4D), LTC4 (Fig. 4E), and ECP (Fig. 4F) levels in the three tissues (*P* < 0.05) while quail egg treatment was able to significantly inhibit the release of these eosinophil related mediators (*P* < 0.05) and tissues recovery to reached normal levels.

### Oral quail egg treatment reduced the generation of pro-inflammatory markers in tissues

**Figure 5:**
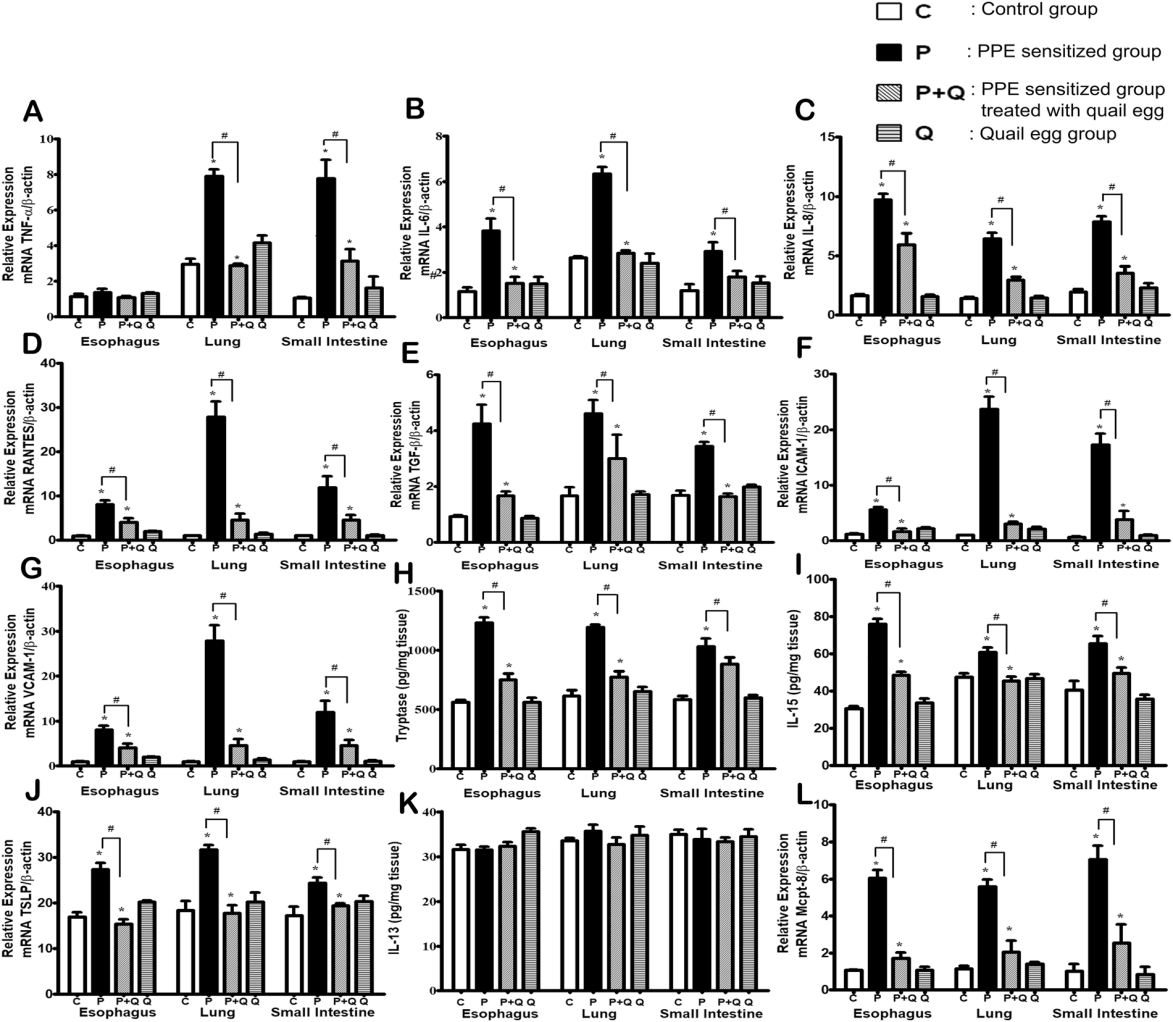
Oral quail egg treatment reduced the generation of pro-inflammatory markers in tissues (n = 5). (**A**) TNF- alpha, (**B**) IL-6, (**C**) IL-8, (**D**) RANTES, (**E**) TGF-β, (**F**) ICAM-1, (**G**) VCAM-1, (**H**) Tryptase; (**I**) IL-15, (**J**) TSLP, (**K**) IL-13, (**L**) mMCP-8. Results are expressed as mean ± SEM. *P < 0.05 as compared to C group, ^#^*P* < 0.05 as compared to P group.

Tissue inflammatory response in EoE is strongly linked to other allergic inflammatory diseases showing similar responses in the induction of inflammatory cytokines, chemokines and adhesion molecules^25,26^. Therefore, we detected the mRNA levels of pro-inflammatory cytokines and chemokines (TNF-α, IL-6, IL-8, RANTES, TGF-β) and mRNA levels of adhesion molecules (ICAM-1 and VCAM-1) in the oesophagus, lung, and intestine tissues. The results indicated that quail egg could significantly decrease the levels of pro-inflammatory cytokines and adhesion molecules induced by PPE (*P* < 0.05, Fig. 5A–G) in all three inflamed tissues, however we did not find significant difference change in the mRNA levels of TNF-α of oesophageal tissue (Fig. 5A).

In addition, the same result was observed with serum and splenocyte mediators and cytokines released, quail egg administration significantly reduced the elevation of tryptase, IL-15, and TSLP levels (*P* < 0.05, Fig. 5H–J) whereas no change was found in the IL-13 levels (Fig. 5K). In the EoE disease pathogenesis, basophils also play key role^27^, we found the level of mMCP-8 in PPE sensitized and challenge (P) mice group was significantly increased (*P* < 0.05) while the elevation level of mMCP-8 could be decreased following oral quail egg treatment (Fig. 5L).

### Oral quail egg treatment reduced the expression of PAR-2 and NF-κB p65 in tissues and promoted the cure of EoE like disease therapy

**Figure 6:**
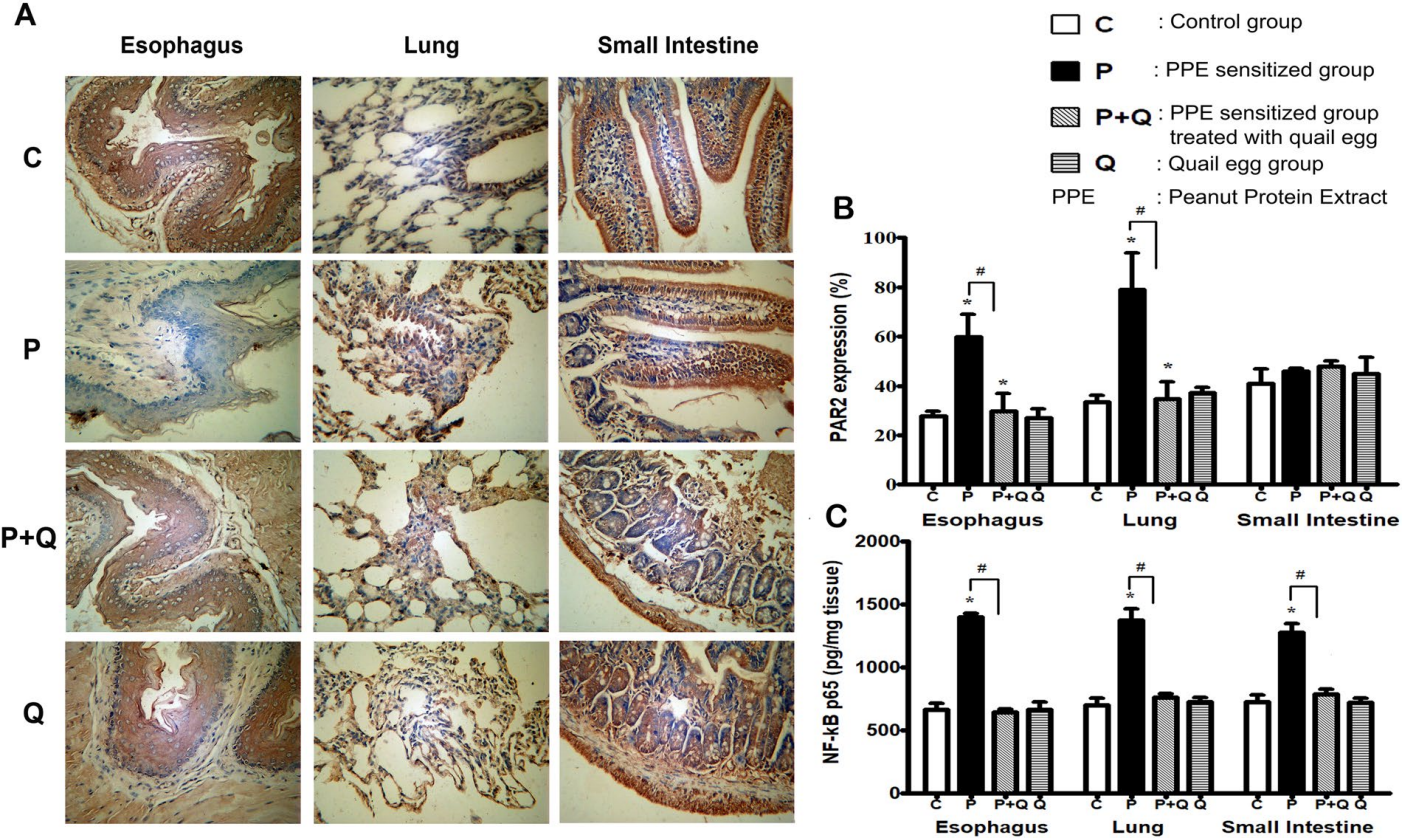
Oral quail egg treatment reduced the expressions of PAR-2 and NF-κB in tissues and promoted the cure of EoE like disease therapy (n = 5). (**A**) Immunohistochemistry of oesophagus, lung, and small intestine mice organ tissues. Scale bar 100 μm at 400×, (**B**) Immunohistochemical score analysis, (**C**) NF-κB p65 phosphorylation. Results are expressed as mean ± SEM. *P < 0.05 as compared to C group, ^#^*P* < 0.05 as compared to P group.

A study by Vergnaud and Bruttman found that quail egg had inhibitory effect on human trypsin activity which led to inactivation of PAR-2 receptors^10^. Therefore, we did further investigation of the effect of quail egg treatment on PAR-2 receptor activation in our EoE like food allergy murine model. Using immunohistochemistry method, we observed qualitative and quantitative assessment of the expression of PAR-2 in oesophagus, lung and small intestine tissues. Compared to control group, results indicated that following repeated PPE challenge, the expression level of PAR-2 was significantly increased in P group oesophagus (59.75 ± 18.55%) and lung (79.00 ± 39.03%) tissues (*P* < 0.05) whereas the expression of PAR-2 in P + Q group and Q group showed no significant difference (Fig. 6A-B). Moreover, there was no change in PAR-2 expression in small intestine of each group.

PAR-2 is able to activate the downstream NF-κB signalling pathway and play an important role in inducing inflammation^28^. The phosphorylation of p65 is the main key of the NF- κB signalling pathway activation^29^. Our results showed that after PPE stimulation, p65 (Ser536) phosphorylation level in P group was significantly increased (*P* < 0.05), whereas daily quail egg treatment (P + Q group) significantly inhibited this phosphorylation process (*P* < 0.05) which reached the same level as control group (*P* < 0.05). Mice treated with quail egg alone (Q group) did not affect the activation of p65 (Ser536) (Fig. 6C).

## Discussion

Study of Feeney *et al*., who compared the inhibition effect of 12 bird eggs, discovered that only quail egg, and particularly its ovomucoid, could be act as a potent serine protease inhibitor^9^. In 1971, Liu *et al*. found that another protein fraction of quail egg in 40 kDa molecular weight, called ovoinhibitor, also possessed an anti-trypsin like serine protease inhibitor^30^. This ovoinhibitor glycoprotein was also acting as inhibitor on chymotrypsin, subtilisin (bacterial enzyme isolated from *Bacillus subtilis*) and fungal proteinase isolated from *Aspergillus oryzae*)^30^. Previous studies suggest that the white ovoinhibitor of quail egg acts as an inhibitor of the type arginine-trypsin^31^ while quail egg ovomucoid acts as a serine protease inhibitor which is likely placed at amino acid position 162 close to the glycine binding site^9^. Furthermore, quail egg ovomucoid role is based upon its strong anti-trypsin functions as a potent auto serine protease inhibitor, with its active site is likely located on the side chain of Arg^89^ or Arg^90^ within domain II peptide fragments^30^. In the body’s physiological normal condition, the proteases and their natural inhibitors are in equilibrium state^32^. While during allergic disease progression, this equilibrium state is disturbed by the activation of immune cells as well as mast cells, eosinophils, basophils, and others cells which act as potent effector cells in response to hypersensitivity. These effector cells are able to release huge numbers of proteases as well as tryptase, and further contribute in irreversible tissue injury and inflammation^33^. PAR-2 is one of the members of the G protein coupled receptor family and widely distributed in tissues. It can be activated by serine proteases^34^ which acts as key factor to trigger inflammation^35,36^. A study found the link between PAR-2 polymorphism and the development of allergic disease in atopic Korean children^37^. Using PAR-2 specific inhibitors can effectively inhibit the development of airway hyper-responsiveness and inflammation in asthmatic mice^38^ while this inhibition functions can also be observed in other allergic inflammation disease in which using PAR-2 inhibitors can alleviate the symptoms of mice model atopic dermatitis^39^. EoE is recognized as a chronic allergic disease associated with inflammation. Daily administration of quail egg may be an effective strategy for the treatment of allergic diseases without side effects.

We established that PPE alone could not significantly activate eosinophils, hence we combined PPE oral allergen challenge^21,22^ and PPE skin challenge^40^. The aim of these two ways of allergen contacts was to enhance the activation and accumulation of eosinophils. In this study, we established EoE like food allergy model in Balb/c mice. Using this murine model, PPE sensitized and challenged mice showed a significant weight loss or other responses of systemic allergic reactions as well as hypothermia, an increase of scratching behaviour, and other hypersensitivity allergic reactions. In addition, it also showed the response on local allergic symptoms such as diarrhoea and dermatitis. Meanwhile, this murine model also showed the elevation of eosinophil numbers accumulated in their oesophagus, lung, and small intestine tissues as well as the elevation of disease related chemokines, cytokines, or other mediators, the promotion of allergen specific IgE and IgG1, suggesting the pathogenesis of EoE like food allergy disease is similar to EoE animal model food allergy established by Rajavelu *et al*.^21^. Daily oral quail egg treatment could effectively inhibit EoE like food allergy disease progression evidenced by the reduction of allergic reaction, allergen specific antibodies levels, spleen cells EoE related cytokines, eosinophil accumulation, and other inflammatory responses.

We acknowledged quail egg treatment was likely to act as a dietary treatment rather than drugs and most of oral supplements tended to have shorter biological half-life which was between 3 and 4 hours^41^. Therefore, we not only conducted daily oral administration of quail egg but also modulated the drinking water of mice with given continuous access to quail egg for functional study of treatment. Although it had already been indicated that the anti-allergic effects of quail egg is derived from its ovomucoid and ovoinhibitor, but it is only one *in vitro* study that used pure extract of quail egg ovomucoid^10^ while all previous clinical human studies involved administration of whole quail egg to allergic patients instead of quail egg pure ovomucoid extract^12–14^. In addition, new proteomic study successfully identified 29 new protein spots representing 10 different kinds of proteins in quail egg white, where some of found proteins were belonging to SERPIN (serine proteinase inhibitor) family^42^, indicating that quail egg anti allergic agents is not merely coming from its ovomucoid and ovoinhibitor. Furthermore, nutrients contents in quail egg yolk likely also play an important role as anti-allergic agents. Previous studies observed that quail egg is richer in vitamins and minerals as compared to other bird eggs^12,43^. Among these vitamins and minerals contained in quail egg, we think the anti-allergic effects may emanate from its vitamin E. Tolik *et al*. revealed that quail egg yolk contained five times higher vitamin E as compared to other bird eggs^43^ and several studies had indicated the role of vitamin E in the treatment of allergy such as suppressing neutrophil migration^44^, inhibiting IgE production^45^, and also modulating the development of Th cells immunomodulatory^46^. Therefore, we conducted this present work using whole quail egg as pre-experiments to elucidate the role of quail egg in the term of allergy therapy.

Meanwhile, it is largely known that quail egg itself contained egg allergens which also may act on immune pathway regulation to provide benefit in the occurrence of allergy reactions. However, although in this study, we also found that quail egg oral treatment was able to induce a significant augment of quail egg specific IgE on the day 35, we also found a significant reduction of PPE specific IgE and IgG1 in peanut allergens sensitized mice treated with quail egg. The significant elevation of quail egg specific IgE was diminished later on, indicating the development of tolerance to quail egg allergens. The production of IgE antibody against quail egg in this mice group was not surprising as quail egg white contained many described allergens and the mice used in this experiment all received an adjuvant to promote the response. Interestingly, PPE triggered robust peanut allergens specific IgE and IgG1 in sensitized mice while quail egg triggered only a moderate title of quail egg specific IgE and IgG1. This phenomenon probably relates with the route of allergen exposure since it has been described that cutaneous exposure is prone to develop allergic immune response while oral exposure tends to develop tolerance^47^.

The significant effect of quail egg on modulating immunoglobulin levels, especially IgE, contrast previous observations that administration of homogenate quail egg had no modification of total plasma IgE in serum of allergic rhinitis subjects^14^. While it is difficult to draw conclusions by comparing effects of quail egg in mice to a proprietary blend of quail egg in healthy volunteers with low total IgE where this study dealt with allergen sensitized murine model induced to have high levels of IgE. However, the study of Truffer in allergic asthmatic subjects supported our findings^13^. In the study, he found that quail egg therapy led to a sudden rise in IgE in serum of patients observed during the course of quail egg therapy after about one month followed by an appreciable decrease starting with months 2 or 3^13^, indicating the ability of body to develop tolerance to quail egg. However, the consequences of quail egg treatment in serum total IgG1 have not been described previously and appear to be minimal. Overall, these indicate that the protein fractions, such as ovomucoid, contained in quail egg may be different from the already identified allergen proteins, which may be safe therapeutic approach for treating allergic disease and may act on immune pathway regulation to provide benefit in allergy. Moreover, due to the fact that quail egg has been known by its inhibition role on protease activity, we performed further deep research, focusing on anti-allergic mechanism effects of quail egg, through its role in inactivation tissue inflammatory responses by blockade the activation of PAR-2 and modulating NF-kB downstream signalling pathway.

**Figure 7:**
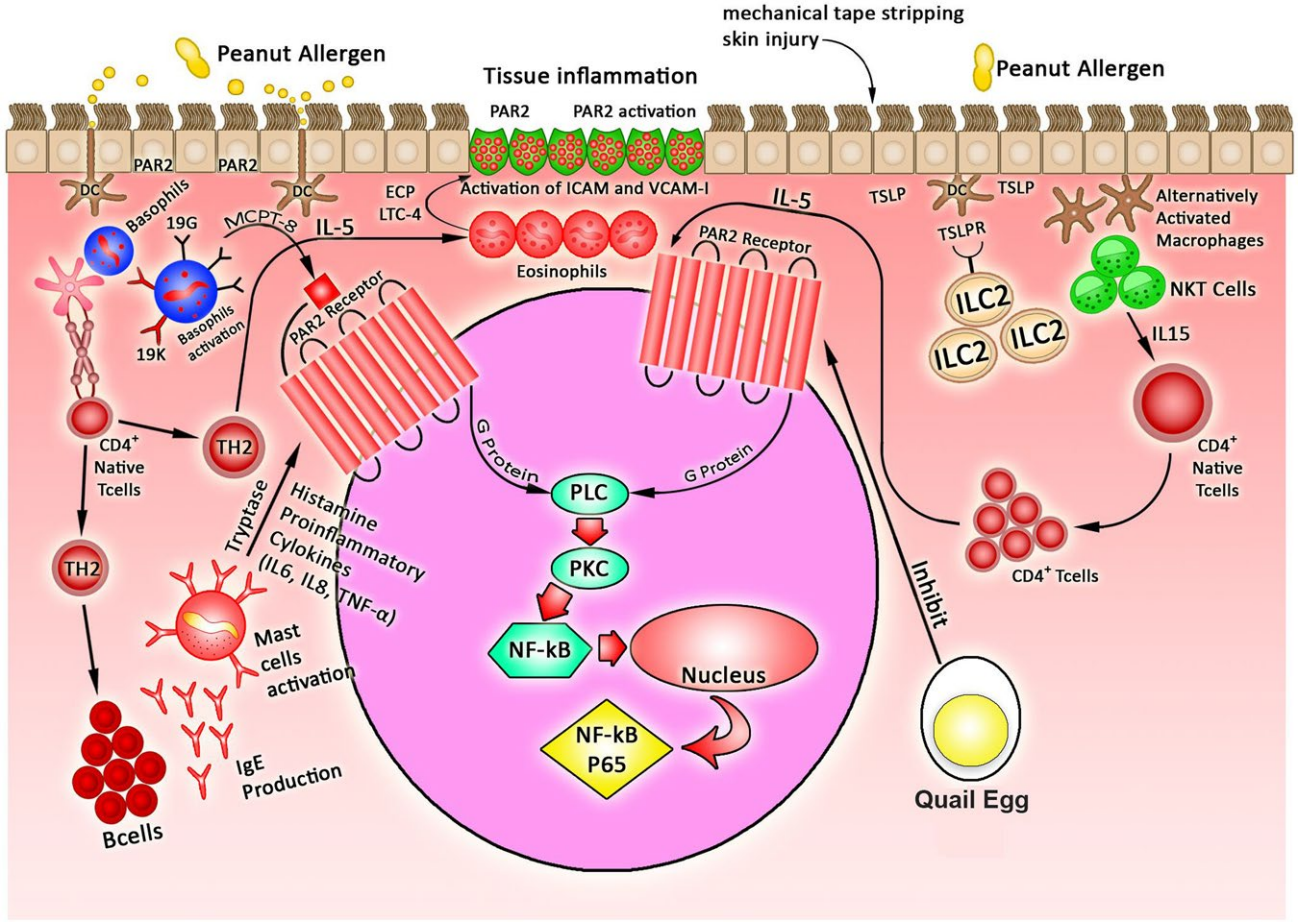
Schematic diagram of the mechanism of anti-allergic and inflammatory effect of quail egg on the food allergy induced EoE like disease alergic reaction.

Based on the results of our study, the effects of quail egg on the treatment of EoE like food allergy disease can be concluded in Fig. 7. Peanut allergens, as a trigger in the experimental of EoE, are captured by dendritic cells (DCs) through epithelial barrier disruption. Thus, peanut allergens induce the activation of mature DCs and facilitate them to migrate to regional lymph nodes where mature DCs present captured allergen epitopes to cognate T cells. Food allergens can then penetrate also in the skin, bind to antigen-related receptors and activate epithelial cells to produce pro-inflammatory cytokines responsible for the recruitment and activation of inflammatory cells including eosinophils^19,20^. In addition, tape stripping skin mechanical injury, which is conducted before PPE skin sensitization, allows the breaking of the skin barrier, the entry of peanut allergens which will next be captured by skin DCs, and thus induce the activation of ILC2 to release TSLP^48^. In the presence of epithelial injury, TSLP production is elicited from epithelium^27,48^. TSLP supports Th2 differentiation through promoting the secretion of Th2 cytokines to epicutaneous sensitization through polarizing skin DCs^48^. In addition, peanut allergens also induce NKT-cells released IL-15 leading to the increase production of macrophages and DC the inflamed tissues in the experimental EoE^21^. In the condition of high level of IL-4 produced by Th2 and ILC-2, the IgE in presence of IL-4 leads to up-regulation of FcεR1 density on the surface of mast cells^49^, while IgG up-regulates FcγR1 on mast cells^50^. The activation of mast cells and basophils leads to the release of pro-inflammatory mediators (TNF-α, IL-6, and IL-8), which contribute to the local inflammatory responses^20,24^ and promote the development of allergic inflammation through the activation of PAR-2 mediated NF-kB transduction pathway^28,29^. PAR2 activation, through coupling with G proteins, facilitates variety of signaling cascades including phospholipase C (PLC) activation which supportes Ca^2+^ mobilization and then triggers NF-kB p65 transduction pathways and promotes the influx of eosinophils^28,29^. Thus, in response to allergic stimuli, eosinophils are attracted to the inflammatory sites by the orchestration of Th2 and cytokines (IL-5), adhesion molecules (ICAM-1, VCAM-1), and chemokines (eotaxin-1, RANTES)^24^. In addition, IL-15 cytokines released from NKT cells are also able to induce CD^+^ T cell activation produced eosinophil active cytokines IL-5 regulated by STAT5. IL-15 cytokines also induce eotaxin in the esophageal epithelial cells that attractes eosinophils into the inflamed tissues epithelial mucosa from the blood^21^. Besides, IL-5 cytokines synergize with eotaxins to enhance mobilization of eosinophils into the tissues following allergen exposure, and thus trigger eosinophils to release lipid mediators (LTC4) and ECP^24^. Eosinophils also derive TGF-β to induce fibrosis at the inflammatory foci, contributing to tissue remodeling and inflammation^24^. Quail egg can act as serine protease inhibitor which could be able to blockade the activation of PAR-2. Overall, our results indicated that quail egg was able to significantly down-regulated PAR-2 receptor activation, inhibited the phosphorylation NF-kB downstream signalling pathway, inhibited the development of inflammatory responses, as well as EoE related inflammatory cytokines (IL-6, IL-8, TNF-α), adhesion molecules (ICAM-1, VCAM-1), and chemokines (eotaxin-1, RANTES), eosinophils related mediators (ECP, LTC-4, TGF-β). Quail egg could also suppress allergic inflammatory process by inhibiting the polarization of Th2, ILC2, and NKT cells, and the levels of Th2 cytokines (IL-4, IL-5), ILC2 (TSLP), and NKT cells (IL-15) were significantly decreased after treatment, but this treatment did not affect the level of IL-13. This treatment was also able to up-regulate the production of IL-10. The decrease in the Th2 and ILC2 cytokines directly inhibited the production of PPE specific IgE and IgG1. In addition, the levels of quail egg specific IgE was lower than PPE specific IgE levels. Decreased IgE and IgG1 levels following oral quail egg treatment also affected the activation of mast cells and basophils during allergic disease progression. We also found that quail egg treatment was able to bring the significant reduction of mast cell mediators (histamine, tryptase) and basophil mediator (mcpt-8) levels. And all these suppressing effects brought to food allergy induced EoE like disease symptoms attenuation.

In summary, this study suggests that oral quail egg treatment can be used as reliable dietary supplement and safe therapeutic approach to treat EoE like food allergy disease and improve the lives of patients with EoE. Further studies on the active substances derived from quail egg mechanisms of action underlying the observed anti-allergic effects are needed and will be critical to identifying the therapeutic potential of quail egg.

## Materials and Methods

### Drugs and Chemicals

The chemicals were obtained from the following suppliers: Imject^TM^ Alum Adjuvant (77161, Thermo Fisher Scientific, Inc., USA); HRP-tagged goat anti mouse IgG1 (ab97240), HRP-tagged goat anti mouse IgE (ab11580), PAR-2 antibody mAb [SAM-11] (ab184673) (Abcam, UK); TranZol RNA Extraction Kit (ET101), TransScript One-Step gDNA Removal and cDNA Synthesis SuperMix (AT311), TransStart Top Green qPCR SuperMix (AQ131) (TransGen Biotech, China); and commercial mouse ELISA kits (eBioscience, Inc., USA). All other chemicals and solvents used in this study were of analytical grade.

### Experimental animals and management

Female Balb/c mice with 7–8 weeks of age were purchased from Weitong Lihua Experimental Animal Technology Co., Ltd. (Beijing, China; No: SCXK(Jing)2016–0001) and acclimatize to their new housing for a week before beginning experimental protocols. Animal experiments employed age-, gender- and genetic strain-matched controls to account for any variations in data sets compared across experiments. Mice were bred and housed under specific pathogen free (SPF) conditions, in the animal laboratory of College of Food Science and Nutritional Engineering, China Agricultural University (Beijing, China). Experimental mice rooms were maintained with temperature of 23 ± 3 °C, relative humidity of 40–70%, light/dark cycle of 12 h, and air exchanges at 15 times/h. Experimental Mice were provided with *ad libitum* access to fresh filtered water and standard rodent diet (moisture, ash, crude protein, fat, crude fibre, calcium and phosphorus) produced by Ke Ao Xie Li Feed Co., Ltd. (Beijing, China) met Chinese Standard GB14924.3–2010 feeding condition requirement, and the limit of detection for aflatoxin was below 20 μg/kg. All experiments were performed under the China Agricultural University Animal Experimental Welfare and Ethical Inspection Committee approved protocols and in accordance with its guidelines. All efforts were made to minimize the suffering of experimental animals.

### Sample Preparation

The crude peanut protein extract sample was prepared from defatted unrefined peanut according to method described by Rezende *et al*.^51^. Protein fixations in the peanut solutions were measured by BCA assay kit.

Normal, commercially available, fresh quail eggs (*Coturnix sp.)* were obtained from a local egg market. Quail egg white and yolk were mixed using mixer (Joyoung Co Ltd., China), freeze-dried and powdered using vacuum freeze-dried (Alpha 1–2 LD plus, Martin Christ, Germany), and then packed and stored at −20 °C.

### Establishment of peanut allergen induced EoE like food allergy disease model food in Balb/c mice

The model was set up as shown in Fig. 1A, seven to eight week old female Balb/c mice (n = 15 mice/group) were sensitized by intraperitoneal injection (2 mg Imject^TM^ Alum Adjuvant (Thermo Fisher Scientific Inc., USA) and 200 μg PPE dissolved in 100 μL PBS) on days 0, 7 and 14 days. At the same time, according to Spergel *et al*.^40^, mice were sensitized by skin contact three times, respectively, on the 1^st^, 21^st^ and 42^nd^ days. The specific methods are as follows: the back hairs of mice were shaved, tape stripped six to eight times using adhesive seal tape mimicking atopic dermatitis like skin lesions, and then applied 1 × 1 cm antiseptic gauze containing 200 µg PPE (dissolved in 50 µL PBS) PPE for a week period, and afterward mice were subjected to intragastric challenge with 2 mg PPE (dissolved in 200 µL PBS) on days 29, 31, 33, 35, 38, 41, 43, 45, 47, and 49 as formerly described^21,22^. Mice were sacrificed 24 h after last PPE intragastric challenge (on the day 50). In the experiment, the control group was not subjected to PPE but only sensitized with 2 mg Imject Alum Adjuvant (Thermo Fisher Scientific Inc., USA) dissolved in 100 μL PBS, merely epicutaneous sensitized with 50 μL PBS, and intragastric challenged with 200 μL PBS.

### Administration of the homogenate quail egg

Administration was done as shown in Fig. 1A, four different groups of Balb/c mice (n = 15 mice/group) were studied; Group 1, control group (C); Group 2, PPE sensitized and challenged group (P); Group 3, PPE sensitized challenged and treated with quail egg group (P + Q); Group 4, non-peanut sensitized and challenged but treated with quail egg group (Q). The amount for quail egg oral administration was followed Integrative Therapeutics (integrativepro.com/allqlear . 800.931.1709) quail egg intake amount which was 84 mg/day^52^. The average weight of human body in China is around 60 kg. The treatment dose for mouse was followed respectively A Simple Practice Guide for Dose Conversion between Animals and Human^53^. The formula of calculation was:

$$\begin{array}{rcl}{\rm{human}}\,{\rm{equivalent}}\,\text{dose}\,(\text{mg}/\text{kg}) & = & {\rm{mouse}}\,\text{equivalent}\,(\text{mg}/\text{kg})\\  &  & \times \frac{{\rm{dosage}}\,{\rm{conversion}}\,{\rm{factors}}\,{\rm{for}}\,{\rm{mouse}}}{{\rm{dosage}}\,{\rm{conversionfactors}}\,{\rm{for}}\,{\rm{human}}}\\ \frac{84{\rm{mg}}}{60{\rm{kg}}} & = & \text{mouse}\,\text{equivalent}\,\text{dose}\,(\text{mg}/\text{kg})\times \frac{3}{37}\\ {\rm{mouse}}\,{\rm{equivalent}}\,{\rm{dose}}\,(\text{mg}/\text{kg}) & = & 17.23\approx 17\end{array}$$

So in this experiment, the dose of quail egg was 17 mg/kg weight body/animal. Quail egg was administered orally in 200 µL PBS on daily basis between days 21 and 49. During challenge period, Balb/c mice were given quail egg oral administration 1 h before each of the ten peanut protein extract intragastric challenges and also given concurrently continuous access to quail egg by feeding water containing 17 mg/L quail egg. For the Q group, we merely conducted the daily oral quail egg administration between days 21 and 49, while for the C group, we administered 200 µL PBS only.

### Clinical Symptom Score

Forty minutes after challenge, mice systemic allergic symptoms (hypothermia and hypersensitivity responses) and local allergic symptoms (diarrhoea, itching and dermatitis) were observed in blinded manner by using a scoring system as formerly described.

The systemic allergic symptoms, hypothermia responses were measured by Beurer FT90 monitoring thermometer (Germany) at 10 min intervals and clinical allergic symptoms were recorded with reference according to the Li *et al*. scoring criteria^54^ with scoring specific criteria as followed: 0 = no symptoms; 1 = nose, lip and eye puffiness, 2 = decreased activities, 3 = dyspnea, cyanosis, no response after challenge or convulsion, 5 = death.

Turn to local allergic symptoms, the diarrhoea symptoms were evaluated according to Rezende *et al*. scoring criteria^51^: 0: no faecal matter or solid state (normal), 1: funicular form, 2: loose stool, 3: slurry, 4: watery state or diarrhoea; the itch responses were evaluated according to the method formulated by Kuraishi *et al*.^55^. In brief, scratching of the rostral parts of the hairless mouse back with hind paws was counted as an itch response. One scratching bout normally comprised of more than three repetitions of mice back stroke scratching movements. A sequence of these scratching movements was measured as one episode of scratching at 10 min intervals. Then, for the dermatitis symptoms were evaluated according to severity of the skin lesions as well as erythema, haemorrhage, excoriation or disintegration, and skin dryness as followed Hashimoto *et al*. scoring criteria^56^: 0: no symptoms; 1: light; 2: mild; 3: moderate; 4: severe.

### Determination of PPE or quail egg specific antibody levels

Balb/c mice blood samples were collected from the mice eyes orbital venous plexus every weeks of challenge period and at day 0, Balb/c mice were bled and their serum were collected as negative control. Mice blood samples were centrifuged for 15 minutes at 4,000×g, 4 °C and serum were collected and stored at −80 °C. Serum PPE or quail egg specific IgE and IgG1 levels were detected by ELISA were measured using ELISA kit according to the manufacturer’s instructions (eBioscience, Inc., USA).

In brief, peanut protein extract or quail egg were pre-coated in 96 well plates for overnight at 4 °C, and then each well was added 100 μL serum plasma and incubated at 37 °C for 2 h, then 100 μL biotinylated antibody was added and incubated 37 °C for 1 h. After washing 3 times with phosphate-buffered saline (pH 7.4) containing 0.1% Tween-20 (PBST washing dilution), each well was added by horseradish peroxidase-conjugated streptavidin (HRP labelled avidin working fluid), incubated at 37 °C for 1 h. After washing 5 times with PBST washing dilution, each well was added 100 μL tetramethylbenzidine (substrate solution) and incubated at 37 °C for 15–30 min under dark condition. In the end, 50 μL of 2 N sulphuric acid (terminate solution) was added to each well, gently mixed, and then within 5 min, the absorbance value of each well was measured at 450 nm wavelength using a microplate reader Thermo Scientific Varioskan Flash (Thermo, USA).

### Detection of allergic mediators and cytokines

Balb/c mice spleens were obtained 24 h following the final intragastric challenge. Splenocyte cells were cultured in RPMI 1640 medium containing neither PPE or quail egg powder for C group, 200 μg/mL PPE for P group, 200 μg/mL PPE and 70 μg/mL quail egg powder for P + Q group, and 70 μg/mL quail egg for Q group under 37 °C, 0.5% CO_2_ incubated condition. After 72 h stimulation, culture supernatants of splenocytes were collected and stored in −80 °C prior to cytokine measurements: Th2 cytokines (IL-4, IL-5, IL-10 and IL-13), ILC2 cytokine (TSLP) and iNKT cell cytokine (IL-15) levels.

In addition to serum plasma allergic mediators, mice plasma were collected in centrifugal tubes which contained heparin, for obtaining plasma histamine, tryptase, and ECP. Besides, for the Balb/c mice tissue lysates (oesophagus, lung, and small intestine), each organ of each group was collected, homogenized and centrifuged at 14,000×g for 30 min, 4 °C and stored in −80 °C before tissue cytokines (IL-5, IL-15, IL-13, TSLP), chemokines (eotaxin-1, ECP),allergic mediators (tryptase, LTC4) and NF- K B p65 levels analysis. All these allergic mediators and cytokines were measured using ELISA kit according to the manufacturer’s instructions (eBioscience, Inc., USA).

In brief, each mediator standards were set and 50 μL standard diluent was added to standard well. 10 μL testing sample was added to testing sample well, and then was followed by added 40 μL sample diluent. Blank well were not added anything. 100 μL of horseradish peroxidase-conjugated streptavidin (HRP labelled avidin working fluid), was added to each well, covered with an adhesive strip, and incubated at 37 °C for 60 min. Each well was aspirated and washed for 5 times with phosphate-buffered saline (pH 7.4) containing 0.1% Tween-20 (PBST washing dilution). 50 μL chromogen solution A and 50 μL chromogen solution B were added, gently mixed, and incubated at 37 °C for 15 min with no light condition. In the end, 50 μL terminate solution (100 μL of 2 N sulphuric acid) was added to each well, gently mixed, and then within 5 min, the absorbance value of each well was measured at 450 nm wavelength using a microplate reader Thermo Scientific Varioskan Flash (Thermo, USA). The detection limits of allergic mediators and cytokines were as followed: histamine (0.1 ng/mL), tryptase (1.0 ng/mL), IL-5 (0.1 pg/mL), IL-4, IL-10, IL-13, IL-15, TSLP, LTC4, eotaxin-1, NF-kB p65 (1.0 pg/mL), and ECP (10 pg/mL).

### Real Time PCR

Total RNA was extracted from Balb/c mice tissues (oesophagus, lung, small intestine) using TranZol RNA Extraction Kit (TransGen Biotech, China), reverse transcribed to generate cDNA synthesis (TransScript One-Step gDNA Removal and cDNA Synthesis SuperMix, TransGen Biotech, China), and SYBR Green Master Mix kit (TransStart Top Green qPCR SuperMix, TransGen Biotech, China) was used for real time PCR. Quantitative real time PCR was performed in triplicate using 96 well plates on the Bio-Rad iCycler iQ system (Bio-Rad, USA). The real time PCR primer sequences were mentioned as below:

*Il-6*: 5′- GTCCTTCCTACCCCAATTTCCA-3′(sense) and 5′-TAACGCACTAGGTTTGCCGA-3′(antisense);

*Il-8*: 5′- TGCTTTTGGCTTTGCGTTGA-3′(sense) and 5′- GTCAGAACGTGGCGGTATCT-3′(antisense);

*Tnfα*: 5′- GATCGGTCCCCAAAGGGATG-3′(sense) and 5′-GGTTTGCTACGACGTGGGC-3′(antisense);

*Icam-1*: 5′-CATCGGGGTGGTGAAGTCTGT-3′(sense) and 5′-TGTGGGGGAAGTGTGGTC-3′(antisense);

*Vcam-1*: 5′-CAAGGGTGACCAGCTCATGAA-3′(sense) and 5′-TGTGCAGCCACCTGAGATCC-3′(antisense);

*Rantes*: 5′-ATGAAGGTCTCCGCGGCACGCCT-3′(sense) and 5′-CTAGCTCATCTCCAAAGAGTTG-3′(antisense);

*Tgfβ1*: 5′TGGCCAGATCTTCACCAG-3′(sense) and 5′GACTGACACGGACTTGAATG-3′(antisense);

*Mmcp-8*: 5′-CCGGAATTCATGTTCCTGCTCCTGGTCC-3′(sense) and 5′-CGCGGATCCCTAGGGTTGTTGCAGGAGTTTCATTG-3′(antisense);

*β-actin*: 5′-TCATGAAGTGTGACGTTGACATCCGT-3′ (sense) and 5′-CCTAGAAGCATTTGCGGTGCACGATG-3′ (antisense).

Quantitative real-time PCR was performed under the thermal cycling conditions included denaturation step at 95 °C for 1 min, followed by 40 cycles at 94 °C for 30 s, 60 °C for 30 s, 72 °C for 30 s, and the final melting curve program with raping rate 0.5 °C/0.05 sec from 55 °C to 95 °C. Using β-actin as the internal control gene, the relative quantitative level of mRNA was calculated by 2^−△△Ct^ method C_t_ is the threshold cycle and △Ct was calculated from test C_t_-β actin C_t_.

### Pathologic Analysis

The oesophagus, lungs and small intestine were fixed in 4% paraformaldehyde. HE staining was performed on paraffin-embedded sections to observe the inflammatory infiltration, tissue damage, villous deformation oedema of epithelium, and other lesions as measured by enumeration of eosinophils per high-power field (HPF).

### Immunohistochemistry

According to the method described by Soslow RA *et al*.^57^, the expression of PAR-2 receptors in oesophagus, lung and intestine tissue was analysed by immunohistochemistry. Tissue was fixed with 4% paraformaldehyde, embedded, sliced de-waxed, added goat serum for 20 min in room temperature, then added anti-PAR-2 antibody mAb [SAM-11] (Abcam, UK) and incubated for overnight at 4 °C. After overnight incubation, sample was washed, added Biotin labelled IgG Antibody, and incubated at 37 °C for 20 min, washed, added HRP-labelled streptavidin based working containing solution (S-A/HRP) and incubated at 37 °C for 20 min, washed, and added DAB solution to colour tissue sections. Haematoxylin stained cells were observed under microscope (Olympus, Japan). Quantitative analysis of the number of positive staining was performed by immunohistochemical score (IHS) based on the German ImmunoReactive score. This method has been shown to approximate data generated from image analysis-based-scoring system^58^.

In brief, the IHS was calculated by multiplying an estimate of the percentage of immunoreactive cells known as quantity score (A) and an estimate of the staining intensity score (B). The formula was IHS = AXB where:

A quantity score was rated on a scale of o to 4 where 0 scored for no staining cells, 1 scored for 1–10% stained cells, 2 scored for 10–50% stained cells, 3 scored for 50–80%, and 4 scored for 80–100% stained cells. In addition to staining intensity score, it was scored on a scale of 0 to 3 in which 0, 1, 2, and 3 represented negative, weak, moderate, and strong staining intensity.

Theoretically, the range of scores could be between 0 and 12. A range of IHS score was divided into 4 groups as follows: 0 for, 1–4, 5–8, and 9–12 groups in which each group represented negative, weak, moderate, and strong immunoreactivity.

### Statistical analysis

Data statistical analysis was performed using two-way ANOVA analysis of variance as parametric test or Friedman test as non-parametric test and calculated GraphPad Prism 5 statistical analysis software (GraphPad Software, San Diego, California). Significant difference was set at *P* < 0 0.05.

### Ethical standards

All procedures performed in this article involving animals were in strict accordance with the China Agricultural University Animal Experimental Welfare and Ethical Inspection Committee approved protocols and in accordance with ethical standard guidelines of China Agricultural University. The manuscript does not contain clinical studies or patient data.
